# Using Protection Motivation Theory to Predict Intentions for Breast Cancer Risk Management: Intervention Mechanisms from a Randomized Controlled Trial

**DOI:** 10.1007/s13187-021-02114-y

**Published:** 2021-11-23

**Authors:** Claire C. Conley, Karen J. Wernli, Sarah Knerr, Tengfei Li, Kathleen Leppig, Kelly Ehrlich, David Farrell, Hongyuan Gao, Erin J. A. Bowles, Amanda L. Graham, George Luta, Jinani Jayasekera, Jeanne S. Mandelblatt, Marc D. Schwartz, Suzanne C. O’Neill

**Affiliations:** 1 Department of Oncology, Lombardi Comprehensive Cancer Center, Georgetown University Medical Center, 2115 Wisconsin Avenue NW, Suite 300, Washington, DC 20007 USA; 2 Kaiser Permanente Washington Health Research Institute, Seattle, WA USA; 3 Department of Health Services, University of Washington, Seattle, WA USA; 4 Department of Biostatistics, Bioinformatics, and Biomathematics, Georgetown University, Washington, DC USA; 5 Washington Permanente Medical Group, Seattle, WA USA; 6 PeopleDesigns, Raleigh-Durham, NC USA; 7 Truth Initiative, Washington, DC USA

**Keywords:** Breast cancer, Prevention, Risk management, Risk-reducing medication, Magnetic resonance imaging (MRI), Protection Motivation Theory

## Abstract

The purpose of this study is to evaluate the direct and indirect effects of a web-based, Protection Motivation Theory (PMT)–informed breast cancer education and decision support tool on intentions for risk-reducing medication and breast MRI among high-risk women. Women with ≥ 1.67% 5-year breast cancer risk (*N* = 995) were randomized to (1) control or (2) the PMT-informed intervention. Six weeks post-intervention, 924 (93% retention) self-reported PMT constructs and behavioral intentions. Bootstrapped mediations evaluated the direct effect of the intervention on behavioral intentions and the mediating role of PMT constructs. There was no direct intervention effect on intentions for risk-reducing medication or MRI (*p*’s ≥ 0.12). There were significant indirect effects on risk-reducing medication intentions via perceived risk, self-efficacy, and response efficacy, and on MRI intentions via perceived risk and response efficacy (*p*’s ≤ 0.04). The PMT-informed intervention effected behavioral intentions via perceived breast cancer risk, self-efficacy, and response efficacy. Future research should extend these findings from intentions to behavior. ClinicalTrials.gov Identifier: NCT03029286 (date of registration: January 24, 2017).

## Introduction

National guidelines present options for breast cancer risk management among women with elevated risk [1]. Women with an estimated 5-year risk of breast cancer ≥ 1.67% and at low risk for adverse events may consider risk-reducing medication (tamoxifen or raloxifene). For high-risk women, these medications reduce 5-year breast cancer risk by 30–55% [2]. Despite the potential benefits, uptake of risk-reducing medication remains low. In the USA, of the 65 million women aged 35–79 without a history of breast cancer, about 10 million are eligible for risk-reducing medication; less than 500,000 use risk-reducing medication [3].

High-risk women with an estimated lifetime breast cancer risk ≥ 20% may also consider annual screening with breast magnetic resonance imaging (MRI) [1]. For these women, annual screening breast MRI is recommended in addition to annual mammography. The limited research on uptake of MRI among high-risk women provides estimates ranging from 9 to 29% [4]. Thus, many high-risk women are not following guidelines for breast cancer risk management or taking full advantage of the interventions available to them.

Despite the low rates of risk-reducing medication and MRI among high-risk women, efforts to increase uptake have been few and have had limited success [5–8]. However, previously tested interventions have not been informed by behavior change theories. To fill this gap, we developed a web-based, breast cancer education and decision support tool for women at an elevated risk of developing breast cancer. This tool was based on Protection Motivation Theory (PMT) [9]. According to PMT, women are most likely to adopt risk management behaviors when they believe that: (1) they are at significant breast cancer risk, (2) risk-reducing medication and/or MRI could be effective at reducing or managing their risk, and (3) risk-reducing medication and/or MRI will be associated with few adverse effects.

A randomized controlled trial compared the PMT-informed intervention to a control arm that directed participants to relevant online health information [10, 11]. One year post-intervention, we found no improvement in uptake of risk-reducing medication due to the intervention. However, among women with ≥ 2.50% 5-year risk for breast cancer, we did observe 4.5-fold increased odds of receipt of breast MRI in the intervention arm compared to the control arm [11]. The null intervention results may be due to the time frame in which outcomes were assessed (1 year following intervention delivery). In addition, risk-reducing medication and breast MRI are both physician-mediated behaviors, in that they require a prescription or a referral.

Given these limitations, we wanted to examine the intervention’s impact on a proximal outcome: intentions for risk-reducing medication and breast MRI at 6 weeks post-intervention. Intentions are an important necessary condition for engaging in recommended health behaviors [12]; thus, examining the intervention’s effects on behavioral intentions would provide important information regarding the overall null effects of the main trial. Additionally, we examined PMT constructs as intervention process variables. Together, these analyses would guide intervention modifications or adaptations.

In the present study, a secondary data analysis examined the direct and indirect effects of the PMT-informed intervention on intentions for risk-reducing medication and/or breast MRI at 6 weeks post-intervention. We hypothesized that (1) the intervention would have a direct effect on intentions for breast cancer risk management, such that women in the intervention arm would report stronger intentions than women in the control arm, and (2) PMT variables would mediate the relationship between study arm and intentions for breast cancer risk management. Our primary outcome of interest was intentions for risk-reducing medication. We also examined intentions for MRI as a secondary outcome.

## Methods

### Participants and Procedures

This two-arm randomized controlled trial (ENGAGED-2, ClinicalTrials.gov identifier: NCT03029286) has been described in detail elsewhere [10, 11]. The trial was approved by the Georgetown University Institutional Review Board (IRB #2015-0687). Briefly, eligible participants were women aged 40–69 and members of Kaiser Permanente Washington, an integrated healthcare delivery system. All women had a normal screening mammogram result between 2016 and 2018, and had an elevated risk of an interval breast cancer per the Breast Cancer Surveillance Consortium (BCSC) 5-Year Risk Calculator [13]. Exclusion criteria included a personal history of cancer, previous referral for cancer genetic counseling, and/or prior genetic testing as documented in the electronic health records.

Women were randomized 1:1 to the intervention or control arm at study sample identification (prior to recruitment). Women randomized to usual care were instructed to review information on the American Cancer Society website related to breast cancer risk, prevention, and cancer screening. The PMT-informed intervention is described below.

A total of 995 women provided verbal informed consent, enrolled in the study, and completed a baseline interview by telephone (intervention = 492, control = 503). Six weeks later, 93% of participants (*n* = 924) completed a follow-up survey (intervention = 459 [93%], control = 465 [92%]) and are included in the analyses presented here.

### Intervention

The PMT-informed intervention has been previously described [10]. In line with PMT [9], the intervention targeted threat appraisals (perceived breast cancer severity and risk) and coping appraisals (self-efficacy, response efficacy, and response cost). Specifically, threat appraisals were targeted through presentation of factual information about breast cancer and personalized 5- and 10-year breast cancer risk estimates. Self-efficacy was targeted through allowing participants to create a tailored question prompt list, and encouraging them to make an appointment with their provider to discuss their questions and concerns. Response efficacy and response cost were targeted through presentation of tailored risks and benefits of risk-reducing medication and breast MRI and an interactive values clarification exercise.

### Measures

PMT constructs and intentions for breast cancer risk management were assessed via self-report at the 6-week follow-up time point.

#### PMT Constructs

##### Cancer Worry

We adapted the 3-item Lerman Breast Cancer Worry Scale [14] to assess worry about getting breast cancer in the future (e.g., “How often did you worry about *getting breast cancer* during the past two weeks?). Participants rated each item on a 4-point Likert scale (1 = “never” to 4 = “almost all the time/a lot”). Items were summed to generate a total score ranging from 1 to 12, with higher scores indicating greater worry.

##### Breast Cancer Severity

Participants rated their agreement with the statement “I believe that breast cancer is severe” on a 5-point Likert scale (1 = “strongly disagree” to 5 = “strongly agree”).

##### Perceived Breast Cancer Risk

Patients estimated their personal risk of experiencing breast cancer in the next 5 years on a scale from 0% (no chance) to 100% (definitely will).

##### Self-Efficacy

Self-efficacy is an individual’s confidence in performing a behavior; in the present study, participants responded to items about self-efficacy of using risk-reducing medication and MRI on a 5-point Likert scale (1 = “strongly disagree” to 5 = “strongly agree”). The four items assessed participants’ confidence in their ability to manage medication side effects, take a pill every day, manage discomfort during an MRI, and have an MRI every year. Items were averaged to generate separate self-efficacy scores for MRI and risk-reducing medication. Total scores ranged from 1 to 5; higher scores indicate higher self-efficacy.

##### Response Efficacy

Response efficacy is an individual’s belief as to whether or not a behavior will avoid a health threat. Participants responded to nine items (three each for tamoxifen, raloxifene, and MRI) on a 5-point Likert scale (1 = “strongly disagree” to 5 = “strongly agree”). Risk-reducing medication items assessed participants’ perceptions that tamoxifen and raloxifene are effective in preventing breast cancer, could significantly improve future health, and are an effective way to reduce breast cancer risk. MRI items assessed participants’ perceptions that MRI is effective in finding breast cancer, could significantly improve future health, and is an effective way to find breast cancer early. Items were averaged to generate separate response efficacy scores for MRI and risk-reducing medication. Total scores ranged from 1 to 5; higher scores indicate higher response efficacy.

##### Response Cost

Response cost is an individual’s perceptions of the downsides of a behavior. Participants responded to three items assessing the costs of risk-reducing medication and four assessing the costs of MRI using a 5-point Likert scale (1 = “strongly disagree” to 5 = “strongly agree”). Risk-reducing medication items included side effects, taking a pill daily, and cost. MRI items included lack of breast cancer risk reduction, discomfort, cost, and potential additional, unneeded tests or treatments. Items were averaged to generate separate response cost scores for MRI and risk-reducing medication. Total scores ranged from 1 to 5; higher scores indicate higher response cost.

#### Primary outcome: intentions for risk-reducing medication

To measure participants’ intentions to use risk-reducing medication, participants rated their likelihood of using tamoxifen in the next year, and their likelihood of using raloxifene in the next year on a 5-point Likert scale (1 = “strongly disagree” to 5 = “strongly agree”). The two items were averaged to create a single score representing intentions for risk-reducing medication.

#### Secondary outcome: intentions for MRI

We measured intentions for MRI by asking participants to rate their likelihood of having a breast MRI in the next year using a 5-point Likert scale (1 = “strongly disagree” to 5 = “strongly agree”).

### Statistical Analyses

Descriptive statistics were used to characterize the sample demographicsand the 6-week follow-up assessment of PMT constructs and behavioral intentions. We described categorical variables using frequencies and percentages, and continuous variables using means and standard deviations. Categorical variables were compared using chi-squared tests; Student *t*-tests were used for the continuous variables. To identify variables to include as mediators in bootstrapped mediation models, we examined correlations between PMT constructs and outcomes at the 6-week follow-up; only potential mediators that were significantly correlated with the outcomes of interest (*p* < 0.05) were included in primary analyses.

Direct and indirect effects of PMT variables on intentions for using risk-reducing medication or MRI were examined using the PROCESS macro for SPSS (Model 4) [15]. The PROCESS macro allows for the estimation of moderation and mediation effects via a bootstrapping procedure. With bootstrapping, effects are estimated based on a large number of bootstrapped resamples (e.g., 10,000 resamples used here) generated from the original data by random sampling with replacement. If the 95% confidence interval (CI) for an effect does not include zero, it indicates the significance of the effect at the 0.05 level. In the present analyses, treatment arm (intervention v. control) was specified as the independent variable. Threat appraisals (cancer worry, perceived breast cancer severity, and perceived breast cancer risk) and coping appraisals (self-efficacy, response efficacy, and response cost) were specified as parallel mediators. Finally, breast cancer risk management intentions (risk-reducing medication and breast MRI) were specified as the outcome variables. Two models were run, one for risk-reducing medication intentions and one for breast MRI intentions.

All analyses were conducted using IBM SPSS for Windows, version 27 (IBM Corp., Armonk, NY, USA).

## Results

The sample was primarily non-Hispanic White (95%), in middle adulthood (*M* = 62 years, range = 40–69), with a college degree or greater (74%) and an annual household income ≥ $70,001 (56%) (see prior descriptions of this sample [10, 11]). The majority of the women were pre-menopausal (93%). About half had a family history of breast cancer (45%) or a prior breast biopsy (45%). Most participants had heterogeneously dense breast tissue (56%) and high (66%) or very high (9%) breast cancer risk.

### Intentions for Risk-Reducing Medication and Breast MRI

Intentions for risk-reducing medication or MRI at 6 weeks were low overall (Table 1). Compared to the control group, the intervention group had significantly greater intentions for risk-reducing medication (*M* = 1.8 versus 1.7, *p* = 0.03). The intervention and control groups did not significantly differ on intentions for breast MRI (*M* = 2.9 versus 2.9, *p* = 0.10).

**Table 1:** Descriptive statistics by intervention group and correlations between mediators and outcome variables at 6 weeks (*n* = 924)

	Intervention	Control		Correlation with behavioral intentions (*r*)	
	(*n* = 459)	(*n* = 465)	*p*-value	Risk-reducing medication	MRI
Mediators					
Cancer worry (*M*, SD)	2.1 (1.69)	2.1 (1.64)	0.979	0.11**	0.14**
Perceived breast cancer severity (*M*, SD)	4.4 (0.87)	4.4 (0.82)	0.863	-0.01	0.04
Perceived 5-year breast cancer risk (*M*, SD)	19.9 (19.48)	25.9 (21.38)	< 0.0001**	0.16**	0.16**
Self-efficacy (*M*, SD)					
Risk-reducing medication	3.3 (0.88)	3.5 (0.83)	0.002**	0.04	0.25**
MRI	4.0 (0.98)	4.1 (0.94)	0.107	0.22**	0.07*
Response efficacy (*M*, SD)					
Risk-reducing medication	3.0 (0.62)	2.9 (0.60)	0.008*	0.11**	0.31**
MRI	3.8 (0.77)	3.7 (0.78)	0.012*	0.32**	0.13**
Response cost (*M*, SD)					
Risk-reducing medication	3.5 (0.77)	3.4 (0.80)	0.078	-0.08*	-0.21**
MRI	3.0 (0.79)	2.9 (0.79)	0.092	-0.21**	-0.05
Outcomes					
Behavioral intentions (*M*, SD)					
Risk-reducing medication	1.8 (0.91)	1.7 (0.91)	0.029*	1	0.26**
MRI	2.9 (1.07)	2.9 (0.98)	0.098	0.26**	1

*M*, mean; *SD*, standard deviation

^*^p < 0.05

^**^p < 0.005

### Correlations Between PMT Constructs and Behavioral Intentions

In bivariate analyses, intentions for risk-reducing medication and MRI were significantly correlated with cancer worry, perceived breast cancer risk, self-efficacy for risk-reducing medication, response efficacy for risk-reducing medication, and response cost for risk-reducing medication (all *p*’s ≤ 0.001) (Table 1). Perceived breast cancer severity was not associated with intentions for risk-reducing medication (*p* = 0.97) or intentions for MRI (*p* = 0.42). Thus, bootstrapped mediation analyses did not include perceived breast cancer severity as a mediator.

### Mediating Effect of PMT Constructs on Intentions for Risk-Reducing Medication

**Fig. 1:**
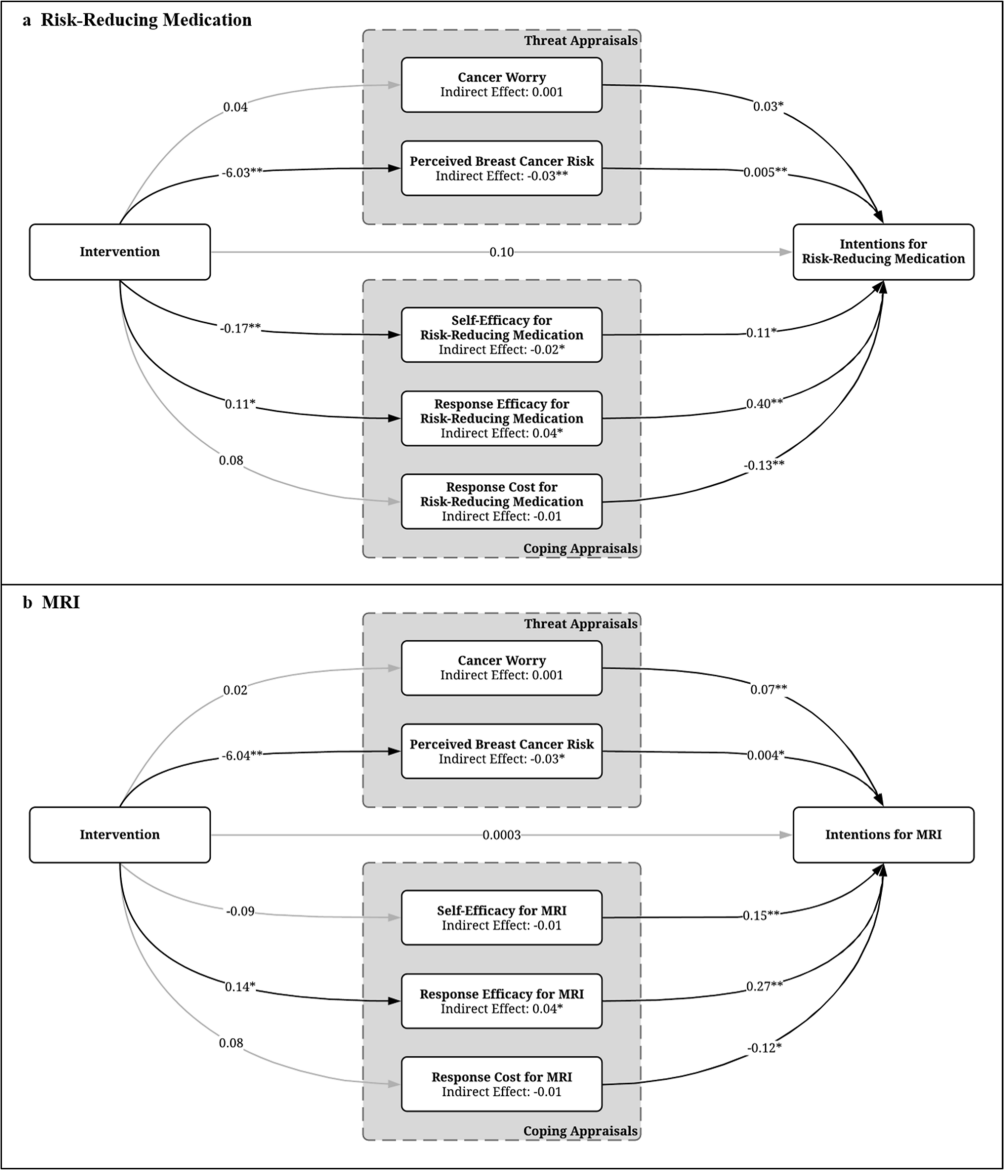
Bootstrapped mediation models examining direct and indirect effects of the intervention on **a** intentions for risk-reducing medication and **b** intentions for breast MRI. All coefficients are unstandardized, and asterisks indicate statistical significance (*p < 0.05, **p < 0.005)

The bootstrapped mediation model predicting intentions for risk-reducing medication explained 16% of the variance in intentions for risk-reducing medication (*R*^2^ = 0.16) (Table 2, Fig. 1a). Neither the total effect nor the direct effect of study arm on intentions for risk reducing medication was significant. There were significant indirect effects of study arm on intentions for risk-reducing medication via perceived breast cancer risk (*p* = 0.004), self-efficacy (*p* = 0.04), and response efficacy (*p* = 0.01). Compared to women in the control arm, women in the intervention arm reported lower perceived breast cancer risk, lower self-efficacy, and higher response efficacy. In turn, perceived breast cancer risk, self-efficacy, and response efficacy were all positively associated with intentions for risk-reducing medication.

**Table 2:** Results of bootstrapped mediation models

	Model 1: intentions for risk-reducing medication (*n* = 901)				Model 2: intentions for breast MRI (*n* = 896)			
	*B*	SE	*p*	95% CI	*B*	SE	*p*	95% CI
Total effect	0.10	0.06	0.115	[− 0.02, 0.21]	0.0003	0.07	0.996	[− 0.13, 0.13]
Direct effect	0.11	0.06	0.055	[− 0.002, 0.22]	0.01	0.06	0.879	[− 0.12, 0.14]
Indirect effects								
Cancer worry	0.001	0.004	0.740	[− 0.01, 0.01]	0.001	0.01	0.864	[− 0.01, 0.02]
Perceived risk	− 0.03	0.01	**0.005**	[− 0.06, − 0.01]	− 0.03	0.01	**0.020**	[− 0.05, − 0.01]
Self-efficacy	− 0.02	0.01	**0.043**	[− 0.04, − 0.004]	− 0.01	0.01	0.192	[− 0.04, 0.005]
Response efficacy	0.04	0.02	**0.009**	[0.01, 0.08]	0.04	0.02	**0.013**	[0.01, 0.07]
Response cost	− 0.01	0.01	0.192	[− 0.03, 0.004]	− 0.01	0.01	0.208	[− 0.03, 0.003]

*B*, unstandardized coefficient; *SE*, standard error; *CI*, confidence interval

### Mediating Effect of PMT Constructs on Intentions for Breast MRI

The bootstrapped mediation model predicting intentions for breast MRI explained 15% of the variance in intentions for breast MRI (*R*^2^ = 0.15) (Table 2, Fig. 1b). The direct effect of study arm on intentions for breast MRI was not significant (*B* = 0.0003, SE = 0.01, *p* = 0.996, 95% C.I. = [− 0.13, 0.13]). Neither the total effect nor the direct effect of study arm on intentions for MRI was significant. There were significant indirect effects of study arm on intentions for MRI via perceived breast cancer risk (*p* = 0.02) and MRI response efficacy (*p* = 0.01). Compared to women in the control arm, women in the intervention arm reported lower perceived breast cancer risk and higher response efficacy. In turn, perceived breast cancer risk and response efficacy were all positively associated with intentions for breast MRI.

## Discussion

We evaluated whether a web-based, Protection Motivation Theory–informed breast cancer education and decision support tool could increase intentions for risk-reducing medication and breast MRI compared to an active control arm. The data presented here demonstrate the important role of threat appraisals, like cancer worry and perceived breast cancer risk, on intentions to engage in breast cancer risk mitigation. Coping appraisals—including self-efficacy, response efficacy, and response cost—were also related to women’s intentions for breast cancer risk management.

We identified three significant mediators of the relationship between study arm and intentions for breast cancer risk management: perceived breast cancer risk, self-efficacy, and response efficacy. Compared to women in the control arm, women in the intervention arm reported significantly lower perceived breast cancer risk at the 6-week follow-up. As women tend to overestimate their risk of breast cancer [16], it is likely that the PMT-informed intervention appropriately decreased perceived risk via presentation of personalized breast cancer risk estimates. Paradoxically, while the intervention led to more accurate risk comprehension, it is also possible that the reduction in perceived risk limited the impact of the intervention on intentions for risk-reducing medication. This may have been particularly salient for women in this study who had not previously received breast cancer risk information in routine clinical care. Thus, participants may have been reassured by the lower than anticipated risk that was conveyed by the intervention.

The intervention group also reported lower self-efficacy for risk-reducing medication at the 6-week follow-up. While there has been little research on the role of self-efficacy in uptake of and adherence to risk-reducing medication, self-efficacy has been shown to play an important role in adherence to other types of medications [17]. Our intervention targeted self-efficacy by encouraging participants to make an appointment with their provider and providing the opportunity to create a question prompt list to use in that appointment. Our relatively short follow-up time frame (6 weeks) may have limited participants’ ability to utilize these strategies. We have previously reported that the proportion of women in the intervention group who had “discussions” with their healthcare providers about risk-reducing medication increased substantially from the 6-week follow-up (5%) to the 12-month follow-up (14%) [11]. Thus, at the 6-week follow-up, participants’ self-efficacy for risk-reducing medication may have reflected the educational components of the intervention, which provided detailed information about tamoxifen and raloxifene. This included the need to take the medication every day and the common side effects for these medications. The intervention’s impact on self-efficacy may be similar to the paradoxical effect seen in prior studies that discussion of the medication regimen and side effects can actually lower self-efficacy for risk-reducing medication [18]. A prior systematic review of adherence to risk-reducing medication noted self-efficacy as a key barrier to adherence [19]. Further examination of its role in initiation could be warranted as well.

Compared to women in the control arm, women in the intervention arm reported greater response efficacy for risk-reducing medication and breast MRI at the 6-week follow-up. Our intervention targeted response efficacy in two ways: presenting tailored risks and benefits of risk-reducing medication and breast MRI, and engaging participants in an interactive values clarification exercise. While a dismantling study would be needed in order to assess the relative effectiveness of these components, it is likely that education about risk-reducing strategies played an important role, given the demonstrated lack of knowledge about risk-reducing medication [20] and supplemental breast screening [21] among women with elevated risk for breast cancer. However, it should be noted that the group differences in mean response efficacy scores were relatively small and may not be clinically significant despite statistical significance.

These indirect effects must be interpreted in light of the null total and direct effects of the intervention on intentions for risk-reducing medication and breast MRI. Although traditional approaches to mediation require a direct effect in order to estimate and test hypotheses about indirect effects, current thinking about mediation analysis does not [22]. Instead, the relationship between two variables (i.e., the total effect) is conceptualized as the sum of many different paths of influence, including indirect effects (i.e., mediation) and/or direct effects. Multiple indirect effects might cancel out, resulting in a null direct effect. In the present study, we observed both a negative indirect effect via perceived risk and self-efficacy, and a positive indirect effect via response efficacy. In other words, the intervention might have both increased and decreased intentions, via different pathways, resulting in no change overall.

Our results support the applicability of PMT to breast cancer risk management. Of the six PMT constructs examined, five were significantly related to intentions for risk-reducing medication and breast MRI. In addition, the direction of the relationships between PMT constructs and behavioral intentions was theoretically consistent. Interestingly, perceived breast cancer severity was not significantly related to intentions for risk-reducing behaviors, and as a result, was not included in the final models. This contrasts with prior meta-analyses examining the relationship between PMT variables and behavioral intentions that have demonstrated a small but significant effect of perceived severity [23]. The discrepancy between the results presented here and prior findings may be due in part to differences in the measurement of perceived severity. In the present study, over 90% of participants “agreed” or “strongly agreed” with the statement “breast cancer is severe” at baseline. The limited range in perceived breast cancer severity may have resulted in a “ceiling effect”, making it difficult to discriminate among subjects reporting high levels of perceived severity.

These results have clinical implications for future interventions in this area. The tendency for women to overestimate their breast cancer risk is well-documented in the literature, and prior risk communication interventions have promoted more accurate breast cancer risk perceptions through the provision of a personalized risk estimates [16]. Accurate risk perceptions are critical to making informed health decisions, but the consequences of this reduction for motivation of health-protecting behaviors requires further consideration. While the current trial reported not only the participant’s 5- and 10-year breast cancer risk, but also the average risk for a woman her age and race, future studies with individuals with clinically elevated cancer risk could place accurate risk perceptions in the context of clinical guidelines.

Promoting medication self-efficacy has become a focus of interventions to promote adherence to oral medications, not only in cancer but in other chronic conditions such as diabetes [24] and arthritis [25]. Unlike control of these chronic conditions, where medication is prescribed to address symptoms, the use of medication for the reduction of breast cancer risk is more preference-sensitive and cannot be tied to an observable metric. Therefore, support for self-efficacy may be even more essential when women are making decisions around initiation of the medication.

Our study had two key strengths. First, we examined theoretical constructs in the setting of a randomized controlled trial. Second, we had a large study sample with a relatively high retention rate; 93% of the baseline participants completed the 6-week follow-up assessment.

Study results must be interpreted in light of some limitations. First, the specified models do not meet the criteria for a “true” test of mediation as the PMT constructs and behavioral intentions were both assessed at the 6-week follow-up time point [22]. Second, the study sample excluded women who had prior cancer genetic counseling or testing, a group most likely to be eligible for and amenable to screening MRI. Third, prior publications have documented that this sample was demographically homogenous [11]. Furthermore, women needed to access online information to participate in the study. Thus, the generalizability of the findings to other ethnic and minority groups or to the underserved is unknown.

In summary, this trial evaluated a novel web-based intervention informed by PMT that provides personalized breast cancer risk communication and decision support. While the intervention did not have a direct effect on intentions for risk-reducing medication or breast MRI, we did observe significant indirect effects of the intervention on breast cancer risk management intentions via changes in perceived breast cancer risk, response efficacy, and self-efficacy. Interventions that address perceived risk and boost self-efficacy and response efficacy may be particularly effective in the context of breast cancer risk management.

## Data Availability

Anonymized data will be made available upon request.
